# Brazilian preliminary norms and investigation of age and education effects on the Modified Wisconsin Card Sorting Test, Stroop Color and Word test and Digit Span test in adults

**DOI:** 10.1590/1980-57642015DN92000006

**Published:** 2015

**Authors:** Nicolle Zimmermann, Caroline de Oliveira Cardoso, Clarissa Marceli Trentini, Rodrigo Grassi-Oliveira, Rochele Paz Fonseca

**Affiliations:** 1 Master Degree, PhD Student. Department of Radiology, Federal University of Rio de Janeiro, Faculty of Medicine; Clementino Fraga Filho University Hospital, Rio de Janeiro RJ, Brazil.; 2 Master Degree, PhD Student. Psychology Faculty, Pontifical Catholic University of Rio Grande do Sul, Porto Alegre RS, Brazil.; 3 PhD. Psychology Institute, Federal University of Rio Grande do Sul, Porto Alegre RS, Brazil.; 4 PhD. Psychology Faculty, Pontifical Catholic University of Rio Grande do Sul, Porto Alegre RS, Brazil.; 5 PhD. Psychology Faculty, Pontifical Catholic University of Rio Grande do Sul, Porto Alegre RS, Brazil.

**Keywords:** cognition, neuropsychological tests, age, schooling, executive function

## Abstract

Executive functions are involved in a series of human neurological and psychiatric disorders. For this reason, appropriate assessment tools with age and education adjusted norms for symptom diagnosis are necessary.

**Objective:**

To present normative data for adults (19-75 year-olds; with five years of education or more) on the Modified Wisconsin Card Sorting Test (MWCST), Stroop color and word test and Digit Span test. Age and education effects were investigated.

**Methods:**

Three samples were formed after inclusion criteria and data analysis: MWCST (n=124); Digit Span (n=123), and Stroop test (n=158). Groups were divided into young (19-39), middle-aged (40-59) and older (60-75) participants with five to eight years of education and nine years of education or more. Two-way ANOVA and ANCOVA analyses were used.

**Results:**

Education effects were found in most variables of the three tasks. An age effect was only found on color naming and color-word naming speed from the Stroop test. No interactions were detected.

**Conclusion:**

In countries with heterogeneous educational backgrounds, the use of stratified norms by education to assess at least some components of executive functions is essential for an ethical and accurate cognitive diagnosis.

## Abstract (pt)

As funções executivas estão envolvidas em uma série de distúrbios neurológicos e psiquiátricos humanos. Por esta razão, instrumentos de avaliação adequados com normas estratificadas por idade e escolaridade são necessárias para diagnóstico dos sintomas.

**Objetivo:**

Apresentar dados normativos para adultos (19 a 75 anos de idade; a partir de cinco anos de educação) no *Wisconsin Card Sorting Test Modificado* (MWCST), teste Stroop de cores e palavras e no teste de Dígitos. Os efeitos de idade e escolaridade foram investigados.

**Métodos:**

Três amostras foram formadas após a análise dos critérios de inclusão e dos dados: MWCST (n=124); Dígitos (n=123) e teste Stroop (n=158). Os grupos foram divididos em jovens (19-39), de meia-idade (40-59) e idosos (60-75) de cinco a oito anos de ensino e nove anos de escolaridade ou mais. Foram utilizados os testes Two-way ANOVA e ANCOVA.

**Resultados:**

Efeitos de educação foram encontrados na maioria das variáveis das três tarefas. O efeito idade foi encontrado somente na velocidade de nomeação de cores e nomeação de cor-palavra do teste Stroop. Não foram detectadas interações.

**Conclusão:**

Em países com cenários educacionais heterogêneos, o uso de normas estratificadas por educação para avaliar, pelo menos alguns componentes, das funções executivas é essencial para um diagnóstico cognitivo ético e preciso.

## INTRODUCTION

Executive functions (EF) are a series of mental processes recruited when automatic responses to problems or environmental demands are no longer sufficient. These processes are basically recognized as inhibition, working memory, and cognitive flexibility, that comprise subcomponents and also lead to higher order cognitive processes, such as metacognition and problem-solving.^1,2^ Many factors are known to affect the course of EF in human life, such as social environment, emotional status, and physical health.^3^ Consequently, EF impairments are highly associated with dysfunction in different life domains, e.g., employment and personal relationships.^4^ Therefore, efforts in the neuropsychology area are necessary to provide assessment tools and performance parameters for assessments.^5^

In spite of the popularity of standardized EF tests in the international literature, few or limited norms exist for the Brazilian population. This topic is especially relevant because normative data from foreign countries cannot be used, since education and culture have an impact on overall cognitive performance. In a study, Ardila, Ostrosky-Solís, Rosseli, and Goméz^6^ described the complex effect of education during aging. The complex effect consisted of a different role of education across the lifespan, which means education does not have the same effect on cognitive performance in different age ranges. However, absence of interaction between these variables was also found.^7,8^ Rosseli and Ardila^9^ presented evidence of a cultural effect even on non-verbal task performance. In view of this evidence, the use of foreign normative data may lead to erroneous diagnosis in clinical and research practice.^10^

Some studies have been published in Brazil, mainly over the last decade. Duncan^11^ published norms for the Stroop color and word test for teenagers in the 12-14 year age bracket, while Klein, Adda, Miotto, Lucia, and Scaff^12^ published norms for elderly in the 60-85 year age range with 2 to 18 years of education. Campanholo et al.^13^ reported normative data for the Stroop test and found age and education effects on a large sample of individuals with ages ranging from 18 to 70 years or older and education from zero to 13 years or more. Ávila^14^ presented normative data for elderly aged 70-73 years for the Modified Wisconsin Card Sorting Test (MWCST). Figueiredo and Nascimento^15^ published normative data for children and adolescents (age range 6-16 years) and adolescents and adults with aged 16-89 years on the Digit Span. In conclusion, the normative studies available for the Brazilian population presented above involve samples with restricted age groups and/or not stratified by years of formal education. For this reason, the aim of the present study was to present normative data for adults aged 19-75 years on the MWCST, Stroop color and word test and Digit Span tests with different educational levels (five to eight years and nine years or more). In addition, age and education effects and interactions between these factors were investigated.

## METHODS

**Participants.** The participants included in this study were community-dwelling volunteers recruited by convenience and contacted at local business companies and community centers. None of the participants received payment for participating in the research. The Institutional Review Board of the university approved the project and all individuals signed an informed consent document. Inclusion criteria consisted of being a native speaker of Brazilian Portuguese, aged 19-75 years and having 5 years or more of formal education. Groups were divided into young (19-39), middle-aged (40-59) and older (60-75) participants with five to eight years of education (low education) and nine years of education or more (high education). After fulfillment of all inclusion criteria, a total of 124 individuals performed the MWCST; 180 the Stroop color and word test; and 160 the Digit Span test. Following data analysis, some participants were excluded for demonstrating lack of comprehension of test instructions (e.g., 35/42 perseverative errors on the MWCST, no correct answers on Digit Span backwards), 42 participants were excluded from the final sample for the MWCST; 38 from the Stroop color and word test; and 15 from the Digit Span task. For this reason, the normative studies involved different sample sizes, as follows: MWCST (n=124); Digit Span (n=123); and Stroop color and word test (n=158).

Individuals self-reporting psychiatric or neurological problems, previous or current abuse of alcohol, cigarettes, illegal drugs or psychoactive medications were excluded. In order to exclude abnormal aging-related cognitive decline, the Mini-Mental State Examination^16^ was administered to individuals aged 40 years and older. All study participants reported no complaints of vision and hearing in order to undergo the tasks. Clinical symptoms of depression were assessed by the Geriatric Depression Scale^17,18^, a scale with yes/no answers. See Table 1, 2, and 3 for sociodemographic, cultural and clinical data of each group.

**Table 1:** Sociodemographic, cultural and clinical characteristics of preliminary normative groups for Modified Wisconsin Card Sorting Test.

Groups by age and education		19-39 years old			40-59 years old			60-75 years old		p
		Five to eight (n=19) Mean (SD)	Nine or more (n=24) Mean (SD)		Five to eight (n=15) Mean (SD)	Nine or more (n=16) Mean (SD)		Five to eight (n=18) Mean (SD)	Nine or more (n=31) Mean (SD)	
Age (years)		33.63 (4.71)	26.67 (6.14)		48.27 (5.97)	47.25 (6.48)		67.00 (4.00)	65.58 (3.70)	<0.001
Education (years)		5.16 (1.21)	13.36 (3.01)		4.93 (1.38)	13.78 (3.90)		4.44 (1.24)	14.13 (3.95)	<0.001
Sex	Male	4	11		3	3		3	8	0.246
	Female	15	13		12	13		15	23	
Reading and writing habits		9.63 (3.35)	12.33 (5.18)		10.40 (3.52)	13.38 (6.68)		11.11 (4.98)	14.83 (4.30)	0.002
MMSE score		*	*		26.00 (2.82)	27.81 (1.68)		24.72 (2.96)	27.71 (2.09)	<0.001
GDS 30		10.95 (3.99)	7.17 (4.72)		8.79 (5.45)	9.47 (4.51)		7.41 (4.62)	6.18 (3.21)	0.015
GDS 15		5.26 (1.96)	3.29 (2.45)		4.27 (2.60)	4.63 (2.24)		3.39 (2.25)	2.50 (1.73)	0.001

* MMSE was not administered to 19-39 year-old individuals.

**Table 2:** Sociodemographic, cultural and clinical characteristics of preliminary normative groups for Digit span test.

Groups by age and education		19-39 years old			40-59 years old			60-75 years old		p
		Five to eight (n=14) Mean (SD)	Nine or more (n=30) Mean (SD)		Five to eight (n=11) Mean (SD)	Nine or more (n=20) Mean (SD)		Five to eight (n=14) Mean (SD)	Nine or more (n=34) Mean (SD)	
Age (years)		33.64 (5.03)	26.93 (5.95)		49.73 (6.05)	49.50 (7.09)		66.43 (4.07)	65.74 (3.73)	<0.001
Education (years)		6.14 (0.94)	13.63 (3.14)		6.18 (1.25)	13.88 (3.49)		5.86 (1.16)	14.18 (3.83)	<0.001
Sex	Male	2	16		2	5		1	10	0.015
	Female	12	14		9	15		13	24	
Reading and writing habits		9.36 (3.27)	12.07 (4.94)		10.82 (4.28)	14.85 (5.85)		11.43 (6.50)	15.21 (4.65)	0.001
MMSE score		*	*		26.36 (1.91)	28.15 (1.56)		26.64 (2.56)	27.47 (2.14)	0.073
GDS 30		11.29 (4.32)	7.23 (4.65)		6.73 (5.01)	8.94 (4.75)		6.10 (4.72)	5.70 (3.27)	0.008
GDS 15		5.43 (2.01)	3.27 (2.37)		3.27 (2.61)	4.40 (2.28)		2.86 (1.91)	2.67 (1.70)	0.001

* MMSE was not administered to 19-39 year-old individuals.

**Table 3:** Sociodemographic, cultural and clinical characteristics of preliminary normative groups for Stroop color and word test.

Groups by age and education		19-39 years old			40-59 years old			60-75 years old		p
		Five to eight (n=20) Mean (SD)	Nine or more (n=30) Mean (SD)		Five to eight (n=16) Mean (SD)	Nine or more (n=23) Mean (SD)		Five to eight (n=21) Mean (SD)	Nine or more (n=48) Mean (SD)	
Age (years)		32.75(4.88)	26.80(6.05)		48.50 (6.04)	48.91(6.76)		67.76(4.36)	67.40(4.30)	<0.001
Education (years)		5.30(1.21)	13.23(3.14)		4.69(1.49)	13.24(3.69)		4.57(1.36)	14.67(4.21)	<0.001
Sex	Male	4	15		4	5		3	7	<0.001
	Female	16	15		12	18		16	26	
	Missing	0	0		0	0		2	15	
Reading and writing habits		10.05(3.20)	11.40(5.00)		10.00(3.63)	14.22(5.99)		9.95(4.70)	14.70(4.99)	<0.001
MMSE score		*	*		26.00(2.75)	27.87(1.68)		24.74(2.76)	27.64(2.14)	<0.001
GDS 30		11.53(4.03)	7.90(4.64)		9.33(5.53)	7.95(4.70)		8.06(4.95)	6.31(3.40)	0.025
GDS 15		5.30(2.17)	3.63(2.38)		4.50(2.68)	3.91(2.33)		3.68(2.38)	2.84(1.64)	0.007

* MMSE was not administered to 19-39 year-old individuals.

**Instruments.** The three tests presented below were administered as part of a larger cognitive test battery of a normative project. The tests were administered and scored by trained research assistants. A double-check method was applied in scoring and data base typing in order to improve reliability of the data presented. All instruments were adapted to the Brazilian Portuguese language following the steps proposed by Fonseca et al.^10^

Modified Wisconsin Card Sorting Test (MWCST). The MWCST first proposed by Nelson (1976) consists of 48 cards containing geometric designs that vary in color, form or number. The original rapport and scoring system were adapted to the Brazilian Portuguese language and culture with help from the author of the adaptation of the traditional WCST for use in Brazil.^19^ Four reference cards are presented to the individuals who must sort the desk cards in color, form or number. However, the sorting rules are not given to participants, such that they must infer by means of examiner feedback whether their strategy is right or wrong. No order of category sorting is required and the participant is told when the rule changes after six correct trials. The deck does not contain ambiguous cards. The scores considered for the task were: categories completed, perseverative errors, non-perseverative errors, and failure to maintain set.

**Digit span.**^20,21^ On this task version the rapport was modified and the number order was pseudo-randomized in order to avoid repetitions. This task consists of two parts. Digits forward is administered first and requires the repetition of digits in the same order presented, while in digits backwards participants must repeat digits in an inverse or backwards order. Part B is administered even if the participant fails in Part A.^15^ Also, Part A must be applied before Part B to enable the assessment of cognitive flexibility components. Correct answers, span scores and discrepancy analysis between parts A and B for accuracy were included in the analysis.

**Stroop Color and Word Test.**^22^ On this task, adaptations were made to rapport, frequency and length of colors, and their respective syllables. The version used consists of a word page (black printed words "pink", "blue" and "green"), color page ("X" letter printed in pink, blue and green) and color-word page with the words presented on the first page with the colors printed on the second page, but colors and words do not match. Participants were asked to read 100 words distributed under five columns equally on a white sheet of paper. Each page should be read in 45 seconds maximum. Errors were considered for scoring, but not discounted from the correct answers, since examiner asked for errors to be corrected when they occurred. Variables considered for analysis were the correct answers for the word, color and color-word pages.

**Data analysis.** Normality of data was analyzed with the Shapiro-Wilk test by stratified groups of age and education. Some variables, although not all, were considered non-parametric after testing. For this reason, parametric and non-parametric tests were conducted and results revealed equivalent directions. Normative groups were compared for years of age, education, reading and writing habits, MMSE score, GDS-30 and GDS-15 by means of a univariate Analysis of Variance (ANOVA); Chi-square analysis was employed to compare groups for sex. Age and education effects were investigated by a two-way ANOVA (MWCST and Digit span) or ANCOVA (Stroop color and word test; with sex as covariate) using post-hoc Bonferroni correction. Effect sizes were calculated for groups with different sample sizes (Cohen's d). Significance level was considered if p≤0.05.

## RESULTS

**Descriptive data from normative samples.** In the MWCST preliminary standardization sample (Table 1), age comparison among groups revealed all groups with high education were younger than the same age-range groups with low education. All groups with the same age-range differed on years of education, and groups of with same education-range did not differ in this regard. The oldest group with high education had lower GDS scores than the younger group with low education (GDS-30 and 15) and the 40-59 years group with high education (GDS-15). Frequency of reading and writing habits was significantly higher in the oldest group with high education than the younger group with low education and the 40-59 years old group with low education.

Table 2 (Digit span data) indicates that the young group with low education was significantly older than the same age-range group with high education. The groups with age range of 40-59 years old with high and low education had equivalent mean age; the same occurred with the 60-75 years group. As expected, the groups with the same age range had significantly different mean years of education, . With regards to GDS-30 score, only the youngest group with low education showed higher scores compared to the oldest group with high education. GDS-15 score of the young group with low education was higher than the same age range group with high education and the two oldest groups. Also, the 40-59 years group with high education showed higher GDS-15 scores than the 60-75 years old group with high education. The frequency of reading and writing habits indicated that the youngest group with low education read less frequently than the 40-59 and 60-75 years groups with high education; whereas the 40-59 years group with low education read less frequently than the oldest group with high education.

Post-hoc analysis of Table 3 with the Stroop test data revealed that young with low education were significantly older than individuals from the same age group with high education. This result was expected since low education has progressively decreased in Brazil with time. Years of education were different among all contrasting education groups. In addition, GDS scores were significantly lower in young with low education compared to 60-75 year-olds with high education. Finally, the oldest group with high education had higher scores for reading and writing frequency compared to low education groups of all ages.

**Age and education effects.** In Table 4, normative data of the three tasks are presented. Age and education effects and effect sizes are available in Table 5.

**Table 4:** Means and standard deviation data for performance on MWCST, Digit span and Stroop color and word test.

Age / Years of education		19-39 years old					40-59 years old					60-75 years old			
		Five to eight		Nine or more			Five to eight		Nine or more			Five to eight		Nine or more	
Variables		Mean	SD	Mean	SD		Mean	SD	Mean	SD		Mean	SD	Mean	SD
**Modified Wisconsin card sorting test**		**(n=19)**		**(n=24)**			**(n=15)**		**(n=16)**			**(n=18)**		**(n=31)**	
• Categories completed		3.74	1.24	5.71	0.69		3.6	1.54	5.19	1.32		3.22	1.59	5.61	0.66
• Perseverative errors		12.05	5.94	2.82	2.63		13.13	5.92	4.04	3.16		13.13	6.07	4.43	4.65
• Non-perseverative errors		5.21	2.46	2.46	3.53		4.47	2.94	1.94	2.29		4.56	3.39	2.31	2.54
• Failure to maintain set		0.79	0.71	0.46	0.83		1.00	0.84	0.44	0.72		0.94	1.11	0.41	0.68
**Digit span**		**(n=14)**		**(n=30)**			**(n=11)**		**(n=20)**			**(n=14)**		**(n=34)**	
• Forward accuracy		6.22	1.47	8.06	1.52		5.86	1.52	7.86	1.59		6.44	1.66	7.47	1.38
• Forward errors		9.78	1.47	7.94	1.52		10.14	1.52	8.14	1.59		9.56	1.66	8.53	1.38
• Forward span length		4.83	0.93	5.71	0.9		4.24	0.94	5.38	1.07		4.64	1.03	5.32	0.68
• Backwards accuracy		3.65	1.02	4.84	1.61		3.43	1.07	4.52	1.43		3.88	1.45	4.68	1.17
• Backwards errors		12.35	1.02	11.16	1.61		12.24	2.14	11.48	1.43		12.12	1.45	11.32	1.17
• Backwards span length		3.17	0.77	3.94	0.81		3.14	0.72	3.86	0.96		3.4	0.86	4.06	0.81
• Forward minus backwards accuracy		2.43	1.28	3.13	1.66		2.18	0.87	3.25	1.83		2.43	1.45	2.79	1.49
**Stroop color and word test**		**(n=20)**		**(n=30)**			**(n=16)**		**(n=23)**			**(n=21)**		**(n=48)**	
• Word correct answers		82.9	30.93	94.4	17.34		73.81	16.1	94.78	22.27		74.21	16.45	89.7	21.13
• Word errors		0.4	0.82	0.13	0.34		0.06	0.25	0.22	0.51		0.05	0.22	0.21	0.54
• Color correct answers		57.75	17.3	69.67	13.79		55.88	9.74	63.78	14.52		51.47	9.38	58.09	10.67
• Color errors		0.4	0.59	0.17	0.46		0.31	0.7	0.17	0.38		0.16	0.37	0.36	0.74
• Color-word correct answers		33.3	11.35	41.73	9.85		28	7.76	37.17	9.98		31.05	8.54	32.88	10.72
• Color-word errors		0.95	1.23	0.57	0.81		0.94	0.99	0.3	0.76		0.53	0.96	0.55	0.86
• Interference score		0.02	8.16	2.34	6.45		-3.35	8.82	-0.34	7.2		0.79	7.02	-1.81	10.62

**Table 5:** Age and education effects on performance of MWCST, Digit span, and Stroop color and word test.

Variable	Age						Education				
	F	p	Post-hoc	Cohen's d	Effect size		F	p	Post-hoc	Cohen's d	Effect size
**Modified Wisconsin Card Sorting Test**											
• Categories completed							85.759	<0.001	H >L	1.805	Large
• Perseverative errors							79.995	<0.001	L>H	-1.92	Large
• Non-perseverative errors							23.770	<0.001	L>H	-0.898	Large
• Failure to maintain set							11.312	0.001	L>H	-0.626	Intermediate
**Digit span**											
• Forward accuracy							26.378	<0.001	H >L	1.025	Large
• Forward errors							26.378	<0.001	L>H	-1.025	Large
• Forward span length							20.126	<0.001	H >L	0.928	Large
• Backwards accuracy							9.227	0.003	H >L	0.629	Intermediate
• Backwards errors							9.227	0.003	L>H	-0.629	Intermediate
• Backwards span length							11.928	0.001	H >L	0.739	Intermediate
• Forward minus backwards accuracy							5.137	0.025	H>L	0.445	Small
**Stroop color and word test**											
• Words correct answers							18.491	<0.001	H >L	0.733	Intermediate
• Words errors											
• Color correct answers	6.942	0.001	Y>E	-0.620	Intermediate		17.627	<0.001	H>L	0.674	Intermediate
• Color errors											
• Color-word correct answers	4.303	0.015	Y>E	-0.548	Intermediate		13.844	<0.001	H>L	0.598	Intermediate
• Color-word errors							4.456	0.037	L>H	-0.303	Small
• Interference score											

Y=19-39 years old; E=60-75 years old; L=low education (5-8 years); H=high education (9 or more years).

## DISCUSSION

The aim of the present study was to investigate the role of age and education in the performance of three executive functions tasks (MWCST, Digit Span, and Stroop color and word test) among adults in the age range of 19-75 years with five years or more of education in a southern Brazilian sample. In addition, preliminary normative data were presented for these tasks. Our data revealed an education effect on almost all variables analyzed. Age effects were observed only for two variables from the Stroop test. No interactions between age and education were found. Implications for clinical and research use of these instruments are discussed below.

**Modified Wisconsin Card Sorting Test.** In the present study, education had a single and main effect on MWCST variables of categories completed (CC), perseverative errors (PE), non-perseverative errors (NPE), and failure to maintain set (FMS). This indicates that age groups had similar performance on this task. This finding differs to the results of previous studies using the modified version and the original Wisconsin Card Sorting Test (WCST). In a meta-analysis review, Rhodes^23^ reported that age had a strong effect on the original WCST and MWCST versions. A study investigating flexibility and working memory contributions to WCST performance found that age-related errors were in fact due to decreased working memory performance in older individuals, not flexibility.^24^ Another study with structural neuroimaging, proposed that age-related increase in PE on the WCST could be explained by a decrease in processing speed, temporal processing, and working memory, which are mediated by the volume of the prefrontal cortex.^25^ However, the role of education in performance on the MWCST has been previously reported. Chan et al. (2003), when using the MWCST, found significant effects of education on groups with two years or less, three to nine years and ten years or more of formal education. The three groups had different performance to each other in CC and total errors. The less educated group had a significantly different performance to the other two groups in NPE and finally for PE, the most educated group had a significantly different performance compared to the two less educated groups. These results suggest that CC may be the variable which best discriminates groups with different educational levels, followed by the NPE and PE. Taken together, these findings may indicate that the cognitive demands required for the MWCST differ slightly to those of the original WCST and that age and education effects may also differ.

**Digit Span test.** Education also showed a single and main effect on Digit Span performance in both forward and backwards forms. A previous study of Hispanic adults in the 16-75 year age range also found no effect of age in the presence of an education effect on the forwards and backwards Digit Span.^26^ Ostrosky-Solís and Lozano^27^ also found education was the stronger predictor of performance on the forward and backward Digit Span in a Spanish-speaking sample. However, the authors also found a cultural effect on these results; another study based on the original North American sample of WAIS-III found independent effects for age and education. An explanation for these findings may be related to the education ranges of each study, as it is known that education level in developing countries are lower than in North America.^27,28^

**Stroop Color and Word test.** Stroop color and word test data demonstrated independent age and education effects on Color and Color-word pages. For both variables, high education individuals performed better than low educated peers, while the younger group performed better than the older group. The results from the Stroop color and word test indicate that the automatic ability of reading is affected only by education, while less automatic abilities, such as naming colors, and inhibition and flexibility required in Color-word page, are affected by both age and education separately. In previous studies, separate age and education effects were found in an elderly sample (60-90 years old)^29^ and in a middle-age and elderly sample (50-90 years old),^30^ and an education effect (with no age effect) was found in a younger sample (18-49 years old).^5^

## Conclusion.

Our findings have some important theoretical implications for the study of sociodemographic variables in cognition, especially with regards to executive functions. As shown by the previous studies described above, education seemed to be more critical than age when comparing group performances, especially for individuals aged up to 75 years old. Moreover, results presented here reinforce the importance for clinicians and researchers to consider age and education variables when discussing neuropsychological performance in order to avoid false positive or false negative errors.

Some limitations of this study are the inclusion of broad age and education ranges, which may have introduced bias, especially to the few age-related findings in the samples. Secondly, the sample size might be considered small, and consequently these results are preliminary. Considering the importance of ecological validity for executive function tests, we suggest that future studies include larger samples with broader age and education variances together with cognitive functional scales or ecological tests. More specifically, oldest-old samples and groups stratified by the quality of education years are relevant demands to be attended in the public Brazilian health and education systems.
